# Corrigendum

**DOI:** 10.1111/mpp.13303

**Published:** 2023-02-13

In Qin et al. (2022), published in Mol. Plant Pathol. 24, 3–15, some of the datasets were not publicly available, and the data availability statement, as well as Supporting Information Table S1 describing the dataset were incomplete. The data were uploaded in the Sequence Read Archive before manuscript submission; however, they were not publicly released because the accession numbers were missing from the data availability statement.

**TABLE S1:** Description of the mRNA and small RNA datasets used in this study.

Description	Library	Read depth	SRA Run	SRA Experiment
*sRNA datasets*				
*B.cinerea*‐tomato 12 h	I12As	76.4M	SRR8074941	SRX4902789
	I12Bs	80.0M	SRR8074948	SRX4902782
	I12Cs	28.5M	SRR8074949	SRX4902781
*B.cinerea*‐tomato 16 h	I16As	85.7M	SRR8074939	SRX4902791
	I16Bs	80.7M	SRR8074938	SRX4902792
	I16Cs	31.4M	SRR8074937	SRX4902793
*B.cinerea*‐tomato 24 h	I24Bs	57.8M	SRR8074936	SRX4902794
	I24Ds	90.7M	SRR8074935	SRX4902795
Mock tomato 12 h	M12As	23M	SRR8074934	SRX4902796
	M12Bs	15.2M	SRR19164837	SRX15231189
Mock tomato 16 h	M16As	41.6M	SRR8074933	SRX4902797
	M16Bs	21.1M	SRR19164836	SRX15231190
Mock tomato 24 h	M24As	21.7M	SRR8074932	SRX4902798
	M24Ds	18.7M	SRR23048732	SRX19002251
*B.cinerea* liquid 16 h	B16As	72.1M	SRR8074931	SRX4902799
	B16Bs	43.6M	SRR8074930	SRX4902800
*B.cinerea* ΔKu70	Ku70‐1s	18.4M	SRR19161653	SRX15228005
	Ku70‐2s	23.8M	SRR19161652	SRX15228006
	Ku70‐3s	15.3M	SRR19161651	SRX15228007
*B.cinerea* ΔKu70 ΔDcl1/ΔDcl2	Dcl1dcl2‐1‐26s	18.2M	SRR19161650	SRX15228008
	Dcl1dcl2‐1‐29s	22.6M	SRR19161649	SRX15228009
	Dcl1dcl2‐1‐30s	17.2M	SRR19161648	SRX15228010
*mRNA datasets*				
*B.cinerea*‐tomato 12 h	I12Am	52.3M	SRR8074958	SRX4902772
	I12Bm	52.4M	SRR8074959	SRX4902771
	I12Cm	24.9M	SRR8074956	SRX4902774
*B.cinerea*‐tomato 16 h	I16Am	52.5M	SRR8074957	SRX4902773
	I16Bm	53M	SRR8074954	SRX4902776
	I16Cm	25.8M	SRR8074955	SRX4902775
*B.cinerea*‐tomato 24 h	I24Bm	54.6M	SRR8074952	SRX4902778
	I24Cm	26.1M	SRR8074950	SRX4902780
	I24Dm	53.9M	SRR8074953	SRX4902777
Mock tomato 12 h	M12Am	13.7M	SRR8074951	SRX4902779
	M12Bm	4.3M	SRR8074946	SRX4902784
	M12Bm	22.4M	SRR19164834	SRX15231192
Mock tomato 16 h	M16Am	23.5M	SRR8074947	SRX4902783
	M16Bm	4.7M	SRR8074944	SRX4902786
	M16Bm	19.8M	SRR19164833	SRX15231193
Mock tomato 24 h	M24Am	18.3M	SRR8074945	SRX4902785
	M24Dm	22.9M	SRR23048700	SRX19002219
*B.cinerea* liquid 16 h	B16Am	22.1M	SRR8074943	SRX4902787
	B16Bm	22.2M	SRR8074940	SRX4902790

**Table:** 

Tissue type = tomato	I, inoculated tomato leaves; M, mock‐treated tomato leaves
Tissue type = fungal mycelium	B, *B. cinerea* liquid culture
Time point of sampling	12, 16, 24 = time point of sampling after inoculation (hours)
Biological replicate	A, B, C, D = code of biological replicate (4 samples were taken, 2 or 3 sequenced)
Type of RNA sample sequenced	s, small RNA fraction; m, mRNA fraction

We provide a revised Table S1 that contains all relevant information about the datasets, read depth and the SRA accession numbers.

The revised data availability statement should read as follows:

The data of this project have been deposited in the NCBI database under Bioproject at www.ncbi.nlm.nih.gov/bioproject/ with accession number PRJNA496584. Raw sequence reads are deposited in the Sequence Read Archive at www.ncbi.nlm.nih.gov/sra/ project SRP166089 under accession numbers SRX4902781‐SRX4902782, SRX4902789, SRX4902791‐SRX4902800, SRX15228005 ‐ SRX15228010, SRX15231189, SRX15231190, SRX19002251 (sRNAs) SRX4902771‐SRX4902780 and SRX4902783‐SRX4902787, SRX4902790, SRX15231192, SRX15231193, SRX19002219 (mRNAs).
